# Architecture

**Published:** 1883-04

**Authors:** Ausburn Towner


### ARCHITECTURE.

AUSBURN TOWNER.

The study of Architecture or the art of building, is one that, either as an occupation of one's leisure, or the business of one's life-time, is something that will return a satisfaction unsurpassed by the pursuit of any other inquiry or calling. It has been an object of interest to mankind from the very beginning, holding a place beside the other physical needs of man. Its relations to civilization are many and manifold, both in the times when it was a necessity and when it eventually became the chief one of the fine arts. You might blot out from the world all painting, and all sculpture, and all knowledge of them. It would be a poor and sad world, to be sure, but man could exist and live in comfort. But you could not blot out architecture, and all knowledge of it, and have *anything* left, save the bare earth beneath you and the blue sky above.

It has been the hard-worked servant, almost the slave of man, and has become his most petted favorite, the object of intense interest in religion, politics, learning and fame. As to its being a hard-worked slave, observe its efforts in times past, and among some rude peoples now, to reach the end wished for by its masters—the tops of adjacent young trees pulled down and tied together, and the sides covered with other branches and leaves; the mud huts, and cabins made of chunks of ice, into which the owner must creep through a hole near the ground, and when once in he is unable to stand erect; the stone cabins and the hemlock shanties; the pits dug from the side-hill or burrowed in the level ground. Are these not the labors of a slave? And yet we know it is the same art that, rising from the mud and rocks and earth, has given us structures so beautiful and harmonious that they are a pleasure to the sight and the memory forever. We know it, because an acute analysis will show us, in any one of these immortal works, that portions of them, in their principles of construction, and in resemblances, come directly down, mayhap from the slender trees bent together, the mud hut or the stone cabin.

Architecture, when it passes from being a necessity, is an educator of great influence and power. Bringing this thought near to us, is it not true that if your neighbor should alter, improve or adorn his house, outside or in; should paint it, or put on a wing, or build a new chimney, or add a portico, that if not yourself, at least your wife would be discontented and unhappy, unless some similar improvement, or some more extensive and pretentious one was made in your own house! That is education, for all improvement is, more or less, education. Improvement as to home, leads to improvement in many other directions, and not the least of all, in culture and refinement, if not to yourself, certainly as to your children and their children.

Twenty-five years ago, there was a little town not many miles from here, that was one of the shabbiest places a stranger ever regretted to enter, or was glad get out of. It was not on the line of any railroad. A two-horse stage dragged itself once a day each way with the mail, and once in a while with a passenger. There was not a house in town that had seen, far less *felt* a paint brush, since the time when the memory of man could be successfully appealed to.

Fences fell down with old age, and the posts rotted. The churches were going to decay, and the school house had to be propped up with flying buttresses, made of old logs. If there was a whole gate in town, it hung by one hinge. It looked as though progress and advance, as they passed, had given the poor little village a kick of disdain and contempt, and left it there to rust, rot and forgetfulness. A railroad was built near it, and a gentleman of means, undeterred by the forbidding appearance, bought a farm near the outskirts. He built a handsome residence, with complete barns and out-houses, and filled it with conveniences and comforts. It was amusing to hear some of the remarks leveled at him by the townspeople. "He has more money than brains"; "What's the use of his coming here to disturb a serenity that has lasted so many years?" "He puts on a great deal of style," and the like. His most bitter reviler was his next neighbor, who rejoiced in the soubriquet of "Bloody Tom."

But it was noticed that "Bloody Tom" very soon began straightening his fences and putting his gates in order; then he painted his house, and how it must have astonished the clapboards! And then he mended his chimneys. Tom's next neighbor, the other way, had to follow Tom's example, of course, and the next beyond him, and the next beyond, until the whole town was encompassed, overhauled and enlivened. You'll not find a brighter place now than this in the whole State of New York, one that is neater or prettier, has better kept churches or a more completely appointed school house. I am not the first one, nor am I alone in attributing this change to the educating influences exerted by the stranger's appearance in town, and building the handsome residence he did. Truly, that bit of architecture was an educator in a place where such education was greatly needed.

A poor hut, into which some immigrant creeps, with its low ceilings, narrow door, and hardly perceptible window, keeps his mind as low down near the earth as his body. Good fortune, in a new country, gives him a better abiding place. As the ceiling goes farther up from the floor, the rooms broaden and widen, and interior or exterior ornament and adornment increases, how does the man also broaden and widen. He has a large breathing place. His mind seems to open like the leaves of a flower, receptive to information as that is to the sunlight and dew, until from being a mere clod, he becomes educated and his children take up the advance where he leaves it, and carry it forward.

These are simple but striking illustrations of the educating influence of architecture, or the art of building, when it passes from being a mere necessity.

Architecture shows to the student the amount of civilization possessed by a nation, people or race, where it exists, and also the *kind* of civilization. This is truthful also of communities. An observer going into a strange place can tell, with a considerable degree of accuracy, a great deal of the character of its inhabitants, by studying the houses as he passes through the streets. Is there public spirit there? Look at the public buildings, the condition of the streets, the manner in which the public parks are kept. Are they hospitable? A house tells for itself, to an experienced eye, whether or not the stranger or friends will be welcome. Religious? Observe the churches, whether they look inviting or forbidding, whether neat and well cared for, or forsaken and neglected. A mercantile community has "business" stamped all over it, as plain to the eye as a farm-house with its barns and various implements indicates agriculture. And so you might go through the whole list of attributes, and, summing them all up, arrive at a a reasonably accurate conclusion.

But the thought has a wider outlook. Here stands before you, let us say, a representative building. It is deep in a shadow made mostly by itself. It looks dark and solemn. The walls are thick and massive, and the columns, capped by the drooping and sleepy-looking lotus flower, or sculptured human face, are ponderous and gloomy. Heaviness seems to be thought a substitute for strength; height for grandeur, and size or mass for power. The idea you get is, that he or they who put up the building, did so, hoping it might remain as long as the earth stands. In the whole structure you see no curved line, nor any other than a right-angle. No graceful arch spans door, or window, or dome. Wall and column, dismal as they are, are thickly covered with carvings and paintings of birds, beasts, fishes and all sorts of creeping things.

Would it require much shrewdness to guess correctly, if we were simply guessing, as to what kind of people they were who erected such a structure ? Would they not be apt to be gross and sensual, not only as to material, but also spiritual things, and yet, with a craving for an immortality, such as they have tried to give their buildings ? It would not be surprising to find that they worshipped animals, and even reptiles. They must be an active, energetic and thriving people, too, else how could such labor be performed as was required to erect structures like the one I have described ? Is it necessary to say that the building is an old Egyptian one, or that the characteristics I have named belonged to that ancient race ?

Here another representative building. Its walls are of pure white marble; the roof has a slant of most gentle slope; the columns surrounding it are proportioned after the ideal form, which, as deities, were worshipped, and the steps have so easy an ascent that a princess could move upwards with hardly a bend of the knee. There is a lightness and airiness about the whole, a harmony in design and execution, and a beauty over all that does not, however, rob it of the idea of strength or permanence. It goes without telling that this is an ancient Greek temple, and from it one could construct the main features of Greek civilization; their lively spirit, their refined culture, their love of ideal beauty, their bravery as soldiers, their activity as citizens.

One more typical structure. Is there not some meaning in it that the least discerning may discover ? The sharply-pointed arches; the slender columns, as though mere earthly support were disdained; the bare, open-timbered roof; the high walls and the lofty ceilings, and the slender spire that shoots upward toward heaven, like the deep sigh from a chest that has been oppressed for years, and at length finds relief. Do not all these appeal to one's sense as having something to do with spiritual aspirations—something to tell of a desire to get away from material and earthly considerations, and nearer to heaven ? Do they not indicate to the student the main features of Christian civilization ?

These are but a few of the many instances where the study of Architecture or art of building returns to the inquirer information curious and valuable. It imparts a knowledge that can have a practical bearing on all hands, and at all times. It is comforting to feel that we know the whys and wherefores of those things by which we are surrounded. The meaning of a particular addition or ornament to a building, the adaptability or applicability of a proposed change, and whether or not it is in harmony with its surroundings, and why. The reason for the rounded or pointed arch, and what the one or the other indicates. Whence the broad portico, the dormer window, the roof sloping almost to the ground, the much-bedecked ceiling, and the plain, fluted or twisted column. For purposes and meanings these all have, and their origin and first use will tell one what they are. Equipped with such information, and much that follows naturally upon it, the capacity of the student for enjoyment and appreciation will be increased an hundred fold, and his life will become happier and fuller.

One who studies nature, we are told, sees in it much that to an uncultured eye is invisible. For him there are "sermons in stones and books in the running brooks." To an equal degree, to one who studies the art of building, will there open almost a new sense. Every building that he sees will have something to tell him. To be sure, he will see satires, and burlesques, and mockeries that will offend, but he will also see true poems in wood, brick and stone. The cathedral will be, to him, a measured chant or a religious epic, the flaunting, domed and much-be pillared capitoline structures, political dramas or parodies, and even the little vine-clad cottage under the hill, hiding itself like the violet, beautiful in its unconscious gracefulness, will be, to him, a pastoral song, singing of love, and home, and happiness.

